# Changes in the proteomic profile of adipose tissue-derived mesenchymal stem cells during passages

**DOI:** 10.1186/1477-5956-10-46

**Published:** 2012-07-24

**Authors:** Emanuele Capra, Riccardo Beretta, Valentina Parazzi, Mariele Viganò, Lorenza Lazzari, Antonella Baldi, Rosaria Giordano

**Affiliations:** 1 Biotrack S.r.l, Parco Tecnologico Padano, Lodi, Italy; 2 Department of Regenerative Medicine, Cell Factory, Center of Transfusion Medicine, Cellular Therapy and Cryobiology, Fondazione IRCCS Ca’ Granda Ospedale Maggiore Policlinico, Milan, Italy; 3 Department of Veterinary Science and Technologies for Food Safety, Università degli Studi di Milano, Milan, Italy; 4 Institute Biotrack S.r.l, Via Einstein 26900, Lodi, Italy

**Keywords:** Adipose tissue-derived mesenchymal stem cells, AD-MSC, S-100A6, Calcyclin, SELDI-ToF, Proteomic

## Abstract

**Background:**

Human mesenchymal stem cells (hMSC) have recently raised the attention because of their therapeutic potential in the novel context of regenerative medicine. However, the safety of these new and promising cellular products should be carefully defined before they can be used in the clinical setting, as. The protein expression profile of these cells might reveal potential hazards associated with senescence and tumoral transformation which may occur during culture. Proteomic is a valuable tool for hMSC characterization and identification of possible changes during expansion.

**Results:**

We used Surface Enhanced Laser Desorption/Ionization-Time Of Flight-Mass Spectrometry (SELDI-ToF-MS) to evaluate the presence of stable molecular markers in adipose tissue-derived mesenchymal stem cells (AD-MSC) produced under conditions of good manufacturing practices (GMP). Proteomic patterns of cells prepared were consistent, with 4 up-regulated peaks (mass-to-charge ratio (m/z) 8950, 10087, 10345, and 13058) through subculture steps (P0-P7) with similar trend in three donors. Among the differentially expressed proteins found in the cytoplasmic and nuclear fractions, a cytoplasmic 10.1 kDa protein was upregulated during culture passages and was identified as S100A6 (Calcyclin).

**Conclusions:**

This study suggests for the first time that common variation could occur in AD-MSC from different donors, with the identification of S100A6, a protein prevalently related to cell proliferation and cell culture condition. These results support the hypothesis of common proteomic changes during MSCs expansion and could give important insight in the knowledge of molecular mechanisms intervening during MSC expansion.

## Background

Human mesenchymal stem cells (hMSC) include a multipotent population with the potential to differentiate into several mesodermal cell types including osteogenic, adipogenic and chondrogenic lineages [1-6]. The regenerative potential of hMSC has recently raised an enormous interest in the novel field of cellular therapy, especially for “no-option” chronic and degenerative diseases. However, the safety of these very promising therapeutic approaches needs to be extensively studied and the knowledge of the critical control points of cell proliferation is still poor. Currently, bone marrow (BM) is the main source of hMSC for both experimental and clinical applications, but this source of hMSC has several limitations: aspiration of BM is an invasive procedure and the frequency and differentiation capacity of hMSC decline with age, thus reducing their therapeutic potential [7]. An alternative source of hMSC is the adipose tissue since its collection is easier and safer than BM. Adipose derived MSC (AD-MSC) can differentiate into other lineages [8] and they have angiogenic [9] and immuno-suppressive properties [10]. hMSC were characterized according to various parameters such as adherence to plastic, differentiation capacity and phenotypic assay based on the expression of specific surface antigen markers. These conventional characterization approaches depicted hMSC as a homogeneous cell population while hMSC appeared extremely heterogeneous by using proteomic and transcriptomic analyses [11]. It has been demonstrated that BM-MSC derived cellular clones with different proliferative capacities have the same cell surface markers by flow cytometry, but they express different protein patterns [12] when studied with different proteomic approaches. Characterization of hMSC using different molecular techniques also revealed that the cells show changes during the expansion which is accompanied by changes in cell markers expression over the culture period [13]. Protein expression maps, created using two-dimensional polyacrylamide gel electrophoresis for continuous subcultures of clonal BM-MSC up to 10 passages, also revealed variation in the proteome during cell expansion [14]. However, these results obtained on MSC clonal populations did not take into account the biological variability between different donors having peculiar molecular profiles. Whether the molecular patterns variations may reflect intrinsic biological modifications during culture or could be shared by different donors is still to be clarified. It is therefore mandatory to monitor AD-MSC inter-individual variability that could limit their application. Herein we report the protein pattern observed in AD-MSC preparations isolated from donors during cell expansion by means of high throughput SELDI-ToF-MS.

## Results

### Cell culture expansion

**Figure 1:**
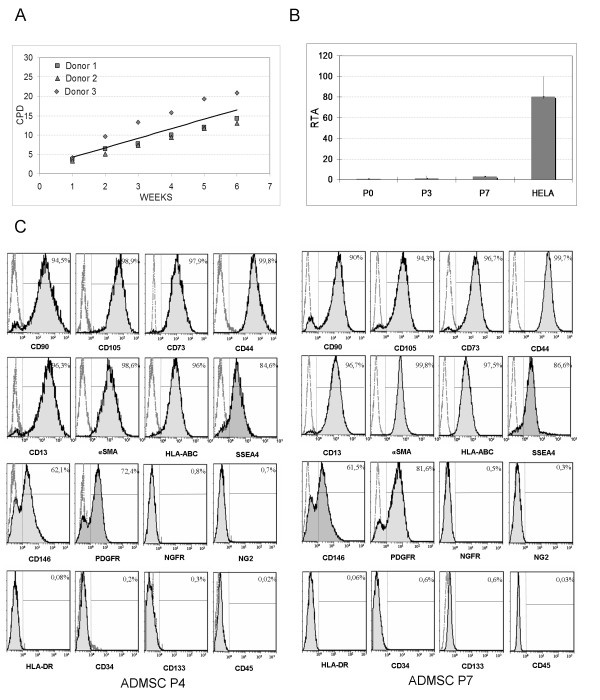
** AD-MSC characterization. A**) Cumulative population doublings of 3 AD-MSC lines. **B**) Telomerase activity in AD-MSC. The RTA was calculated from the different absorbances as follows: RTA = [(As sample-As heated-sample)]/[As Internal standard of the control template x 100]. It is representative of telomerase activity of 3 AD-MSC lines from different donors (median and range). **C**) Representative AD-MSC immunophenotype profile by flow cytometry at passages 4 and 7.

AD-MSC were successfully obtained under GMP conditions from lipoaspirates of three healthy donors. The AD-MSC were analyzed during seven passages and showed a spindle-shaped morphology in confluent-wave-like layers and by flow cytometry they were found to express the typical hMSC surface antigens such as CD90, CD73, CD44, CD105, alpha-SMA, CD13, HLA-ABC and SSEA4; most of the cells were also positive for CD146 and platelet-derived growth factor receptor beta (PDGFRβ). The cells were negative for hematopoietic and endothelial markers (CD45, CD34, CD133; Figure 1) and for HLA-DR, NG2 and NGFR. The immunophenotypic profile was stable during passages (see the representative graphs in Figure 1) for all the donors. The average population doublings for the three AD-MSC lines are shown in Figure 1. The potential of the cells to differentiate into three mesengenic cell lineages was confirmed by their ability to give rise to the adipogenic, osteogenic and chondrogenic commitment after specific differentiation protocols at passages three and five (data not shown). Telomerase activity was assayed in the AD-MSC. The AD-MSC had low telomerase activity which remained approximately unchanged during each passage. In comparison with the human cervical adenocarcinoma cell line HELA (positive controls; Helen Lane, ATCC, CCL-2, Manassas, VA) AD-MSC lines showed a much lower telomerase activity (Figure 1).

### Protein profiling of AD-MSC from different donors

**Figure 2:**
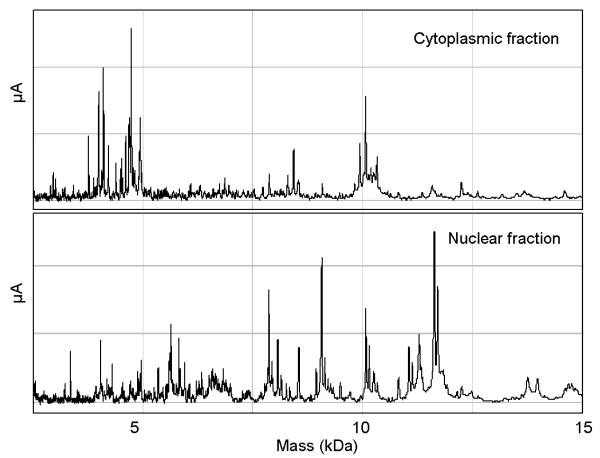
SELDI-ToF spectra of protein extract from different cellular compartments.

**Figure 3:**
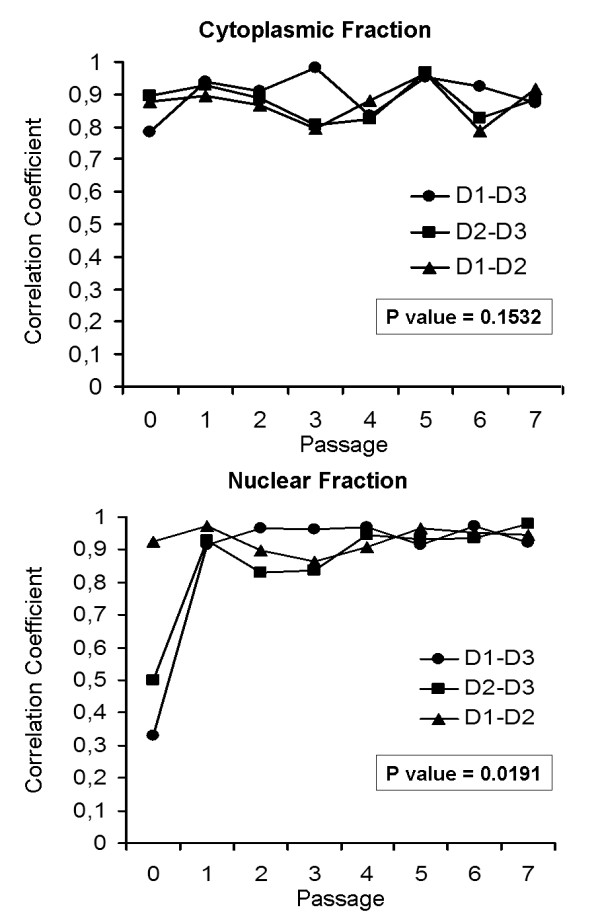
** Donor-to-donor variability.** Variation of the mean correlation coefficients (Pearson’s correlation) between each donor pair (D1: donor1, D2: donor2, D3: donor3) during subculturing passages (P0-P7) for cytoplasmic and nuclear enriched fraction.

All AD-MSC preparations described above were analyzed on a CM10 (weak cation exchange) ProteinChip array. AD-MSC were subjected to fractionation of cellular compartments in order to achieve an enrichment and specific allocation of proteins that vary their expression during subculturing, that results in distinctive spectra exhibiting a variety of exclusive peaks (Figure 2). SELDI-ToF-MS analysis includes longitudinal studies of successive cell culture passages as well as the comparison between donors. Although the profiles appear homogeneous within each cell preparation, inter and intra-individual variations in proteins expression were observed as a consequence of cell expansion process. Donor-to-donor variability was determined by a comparison of mean correlation coefficients during serial culture passage. The results revealed differences between individuals for both fractions representing two different cellular compartments. For the cytoplasmic fraction, the correlation coefficient for donors was between 0.78 and 0.98 and did not change significantly during cell expansion. The nuclear fraction showed a low correlation for each donor in comparison with the other two at passage 0 (0.33 and 0.50), that significantly increased (*P* = 0.0191) in the late passages (0.91-0.98) (Figure 3).

**Figure 4:**
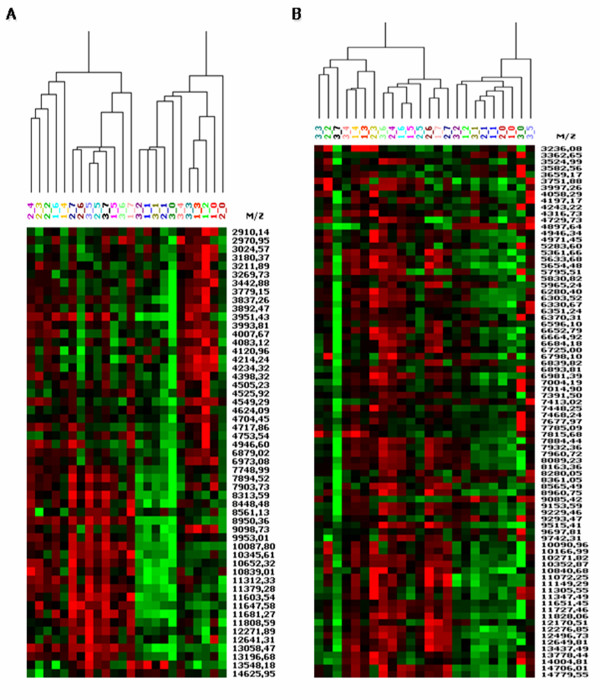
** Graphical representation of cluster analyses of SELDI-ToF mass spectrometry.** Peaks detected in AD-MSC from different donors (1-2-3) during subculturing passages (from 0 to 7) indicated as donor_passage for **A**) cytoplasmic and **B**) nuclear enriched fraction. Red color intensity, high expressed; green color intensity, low expressed.

**Figure 5:**
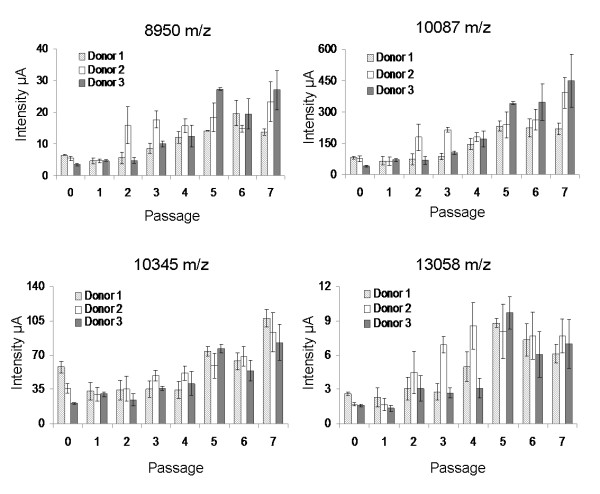
Protein peaks having a similar trend in all the three donors during AD-MSC expansion.

For both fractions the protein profiles revealed a gradual alteration among stages of expansion with samples grouped based on passage number in early and late passages. In particular, cluster analyses of the cytoplasmic extracts split the AD-MSC samples in two groups, P0-P3 and P5-P7. Cluster analysis of the nuclear enriched fraction grouped the AD-MSC from passage P0 to P2, while passage P3, P4 and passage P5, P6, P7 were divided in two subgroups (see Figure 4). Expression profile revealed a total of 53 and 82 peaks detected in the cytoplasmic and the nuclear fractions respectively. In different passages of expansion 41.5% of cytoplasmic peaks and 31.7% of nuclear peaks were differentially expressed (*P* < 0.05) (Table 1; Additional file 1). These protein peaks were however differently regulated during the passages of expansion and between different donors (data not shown), thereby only four of the significant signals from the cytoplasmic fraction showed a common trend and were up-regulated in late passages for all three donors. The peaks with m/z 8,950, 10,087, 10,345 and 13,058 showed significantly increased levels of up to 5-folds during AD-MSC expansion (Figure 5).

**Table 1:** Differentially expressed peaks from different donors during subculturing passages detected in cytoplasmic fraction by SELDI-ToF-MS

**Mass/charge ratio**	**Intensity average**	**p-Value**	**Mass/charge ratio**	**Intensity average**	**p-Value**
11681,3	10,5	0,00510	4505,2	23,6	0,09294
10087,8	180,0	0,00596	4525,9	47,3	0,11063
13058,5	5,0	0,00647	4549,3	49,6	0,11699
10345,6	51,1	0,00827	3892,5	20,6	0,14944
8313,6	25,5	0,01110	12641,3	6,1	0,15640
11603,5	17,1	0,01370	3269,7	7,2	0,16163
8448,5	52,0	0,01433	3993,8	43,6	0,18409
13196,7	4,5	0,01639	4753,5	175,9	0,19162
11379,3	9,9	0,01656	2970,9	24,1	0,19704
8950,4	12,9	0,01801	8561,1	17,1	0,21491
12271,9	13,3	0,02701	7903,7	20,9	0,22520
10652,3	8,4	0,03273	9098,7	13,2	0,22695
3951,4	19,3	0,03273	14625,9	5,0	0,25075
11647,6	14,9	0,03336	4007,7	104,1	0,25748
3211,9	9,9	0,03957	4717,9	91,5	0,27237
11808,6	6,1	0,03995	3442,9	14,0	0,34329
11312,3	5,0	0,04187	4704,4	85,1	0,34450
3779,1	77,4	0,04267	4946,6	94,7	0,40701
9953,0	56,7	0,04599	4214,2	21,6	0,48495
3837,3	12,0	0,04685	4083,1	32,7	0,49240
7749,0	16,6	0,04729	3180,4	11,3	0,51660
4398,3	31,8	0,04909	6973,1	17,1	0,59677
6879,0	24,9	0,06410	2910,1	11,2	0,62746
10839,0	10,1	0,06498	4234,3	41,8	0,68760
7894,5	30,9	0,07050	3024,6	17,2	0,80993
13548,2	3,5	0,07475	4121,0	77,2	0,87368
4624,1	61,5	0,08248			

### Identification of differentially expressed proteins

**Figure 6:**
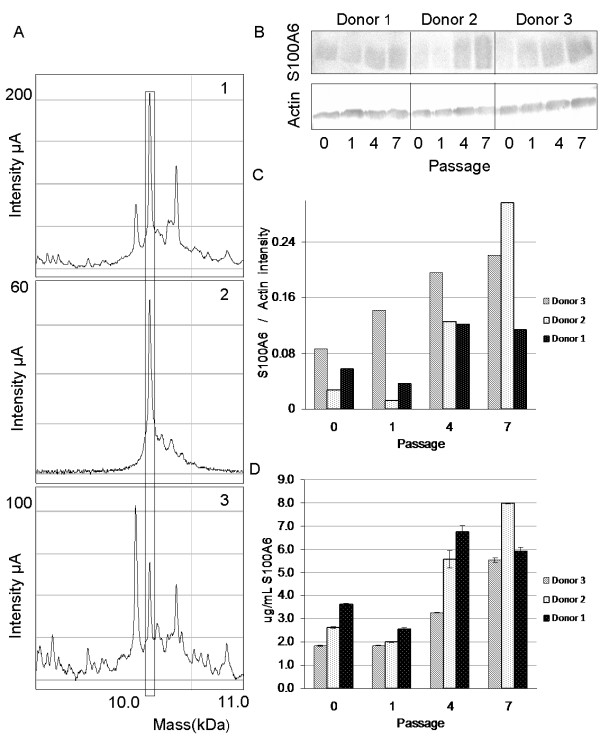
** Validation of S100A6 in cytoplasmic extract of AD-MSC. A**) SELDI-ToF immunoassay of m/z 10,087. Cytoplasmic extract from a pool of AD-MSC at passage 7: 1) spotted on CM10 array; 2) spotted on PS10 array indirectly coupled to anti-S100A6 antibody; 3) eluted from PS10 array indirectly coupled to anti-S100A6 antibody and spotted on CM10 array. The signals enclosed by the rectangle represent the peak at m/z 10,087. **B**) SDS-PAGE/Western blot analysis using anti-S100A6 and anti-Actin antibody of AD-MSC during different passages of cell expansion for different donors. **C**) Western blot densitometry quantification of the corresponding S100A6. Values are expressed as relative intensity ratio between S100A6 and Actin. **D**) Human S100-A6 ELISA quantification. Values are expressed as absolute quantification (μg/ml S100A6).

Based on the marked differences in the intensities of the four peaks at m/z 8,950, 10,087, 10,345 and 13,058 in cells during expansion, we selected these proteins for further characterization. Peaks isolation however resulted positive only for the most abundant peak at 10,087 m/z that was then identified using tandem MS. In particular, analyses of the corresponding chromatographic fraction revealed the presence of several proteins within the peak at 10,180.7 Da closest in mass to that of the target signal and identified as S100A6 protein (data not shown). Previous studies on telomerase-inhibited tumor cell lines identified the peak at 10.1 kDa as the corresponding S100A6 protein [15]. The peak was unambiguously allocated to the S100A6 protein with a SELDI-ToF-MS immunocapture assay by indirect coupling the anti-S100A6 antibody on PS10 protein chip. Spectra from the protein extract bound to the anti-S100A6 array resulted in a signal at 10.1 kDa; conversely the unbound fraction showed a marked reduction in the intensity of the 10.1 kDa peak (Figure 6A). 

### Validation of m/z 10,087 levels by Western blot and ELISA

Up-regulation of S100A6 protein in all donors during cell expansion was firstly validated by immuno-blotting analysis using anti-S100A6 antibody. Late passages of AD-MSC exhibited a significant increase in the amount of S100A6 protein in comparison with passages 0 and 1 (Figure 6B). In particular from passage 0 to 7 the fold increase of S100A6/Actin was 2.5, 10.5 and 2.0 for donor 1, donor 2 and donor 3 respectively (Figure 6C).

Ultimate validation of S100-A6 was performed by Enzyme-Linked ImmunoSorbent Assays ELISA and reported as absolute concentration (μg/ml). Similarly to western-blot analysis, S100-A6 ELISA showed that the level of calcyclin increases during subculturing. ELISA showed a more homogeneous level of calcyclin protein in each passage for all three donors with a mean fold increase of 2.6 (SD 0.8) (Figure 6D).

## Discussion

MSC are a promising tool for cellular therapy and they are used as therapeutic cell products for different clinical applications, predominantly required to address degenerative conditions, organ failure and tissue damage. However, many parameters including heterogeneity of the initial population, donor sources, donor age, and different protocols for cell expansion make difficult the precise molecular definition of primary MSCs. Currently there are no robust approaches for verifying the status of the cells during long-term *in vitro* culture [16-19]. In this context, the need of rigorous standards for characterizing human MSCs is very crucial and several studies are currently ongoing to reach this goal. Recently, new technologies including the analysis of gene expression or proteomic profiling have been suggested as methods to better define MSC preparations [20-22]. In this article we describe the use of SELDI-ToF technology to evaluate the proteomic profile of AD-MSC and examine how this profile changes during growth. SELDI-ToF-MS can detect multiple protein changes simultaneously with high sensitivity and specificity. The high-throughput nature and feasibility in SELDI-TOF-MS experimental procedures made this technology useful for biomarker discovery [23,24], for the characterization of different cell populations (T-cell and fibroblast) [25,26] and with a great potential for clinical perspectives. Other proteomic approaches, such as two-dimensional gel electrophoresis, are more time consuming and less suitable for processing a large number of samples at the same time [12,14]. AD-MSC profiles at different passages were compared using a technique described as differential protein expression mapping, whereby relative expression levels of proteins at specific molecular weights were compared using a variety of statistical techniques and bioinformatic software systems [27]. Our analyses investigated the variation in the proteome profile of AD-MSC during passages.

In our experimental setting, the donor-related variability was minimized by including only healthy subjects with a close range of age. In addition, the isolation and GMP culture procedures were very well standardized and reproducible in order to reduce as much as possible any operation/operator variability. This notwithstanding, the AD-MSC immunophenotypic profile was extremely homogenous during the passages and between the donors, while the proteome profile was very dissimilar. In fact, the hierarchical clustering analyses of protein profile demonstrated considerable inter-individual differences that were consistent with previous data obtained by using other approaches as microarray assays [28]. By fractionating the AD-MSC in the two compartments, cytoplasmic and nuclear, we increased the number of signals detected with a panel of protein peaks differentially expressed in three donors in different passages of expansion. We observed a different trend in the two compartments between donors across different passages. The correlation between protein profiles from different donors was maintained in each passage of expansion in the cytoplasmic fraction. On the contrary, the nuclear fraction showed a poor correlation in one out of the three donors (Donor 3) at P0, while from P1 all the donors had a good correlation with a marked significant increase in protein profiling homogeneity from P5 (Figure 3).

Cell expansion induced protein profiling alteration. Following multivariate analyses the AD-MSC preparations were separated in two groups representing early and late cell culture passages (Figure 4). These changes during MSC long term culture are in line with data reported by other authors and most likely reflect the adaptation to the culture condition [20]. We found that prolonged *in vitro* culture significantly altered the AD-MSC proteomic phenotype, with many of the observed peaks showing significant variation during passages in culture (Table 1; Additional file 1). Although nearly one-third of the total peaks detected in the nuclear fraction was differentially expressed, none showed a similar variation in all the donors during AD-MSC expansion. Protein profiling of cytoplasmic fraction revealed 22 peaks with highly significant changes in expression during passages; among these, only four protein peaks (8,950, 10,087, 10,345 and 13,058 Da) showed a change greater than 2 folds, with a progressive increase from early to late passages for all the donors. Identification of proteins is a major challenge in SELDI-ToF proteomic profiling, however we could identify the most abundant peak at 10,087 as S100A6 protein. Increase of S100A6 during cell expansion was confirmed also by western blot and ELISA. S100 proteins are EF-hand calcium-binding proteins that are involved in the regulation of cellular processes such as cell proliferation, differentiation, migration and apoptosis [29] and their expression is functionally linked to cell cycle progression, cytoskeleton rearrangement [30], exocytosis [31] and other cellular processes. An altered expression of calcyclin has been also seen in different cancers even if its function in tumor development still remains controversial [32,33]. S100A6, also known as calcyclin, modulates signaling pathways by altering the binding potential or availability of calcyclin specific target proteins [34]. Calcyclin is expressed in several cell types with different tissue specificity: it is most abundant in fibroblasts and epithelial cells [35], but it is also found in some neurons, astrocytes [36], smooth muscle cells [37], cardiac myocytes [38] and thyrocytes [39]. Calcyclin expression in hMSC has been seen in hair follicle bulb stem cells where it is up regulated in follicle renewal in response to wounding [40]. Gene expression studies have reported a high level of calcyclin also in BM-MSC [41] and cord blood MSC [42]. Calcyclin is frequently upregulated during proliferation and differentiation and it is induced by different growth factors e.g. platelet-derived growth factor (PDGF), epidermal growth factor (EGF) and serum [43]. Other S100 proteins were seen overexpressed by other factors such as fibroblast growth factor (FGF-2) and transforming growth factor-b (TGF-b) [44]. In line with the finding reported in MSC from other sources, in the present article we found that S100A6 protein levels were overexpressed in AD-MSC during cell expansion. S100A6 variation could be influenced by the *in vitro* propagation condition within other three protein peaks at 8,950, 10,345 and 13,058 Da that were seen to be over expressed during cell expansion. Furthermore, the comparative protein profiling clearly distinguished between short and long term cultured AD-MSC, with cell preparations that seem to become more homogeneous for the nuclear compartment in late passages. Altogether our findings suggest that common response occurs in different donors during expansion, supporting the hypothesis of progressive cellular adaptation to the culture environment or clonal population selection during *in vitro* expansion [20-45]. Nevertheless, if the protein profiling variation in AD-MSC reflects only the long term adaptation to cell expansion or it is also strictly related to the microenvironment conditions still needs to be clarified. Novel markers of MSC that would be linked to specific biological behaviours that could be used as quality controls in cell manufacturing process are needed and SELDI-ToF protein profiling, by reflecting the cell phenotype status, could be a useful tool to monitor and characterize MSC cell during their expansion in different conditions and help translating stem cell technologies into routine clinical applications.

## Conclusions

In conclusion, we observed changes in the proteomic phenotype of AD-MSC following prolonged *in vitro* culture, which were consistent among cells from 3 donors. In particular, the protein with the greatest change in expression during the cell culture was identified as S100A6 or calcyclin. Although the exact nature of observed variation in protein profiles during culture is not known, it could reflect a decrease in the complexity of initial AD-MSC population, resulting in more homogeneous cell type, or the changes may reflect adaptation of cells to their new environment. Advances in proteomic profiling may identify molecular biomarkers to better characterize the phenotypic features of MSC and hence increase the effectiveness of their use in cell therapy*.*

## Methods

### AD-MSC isolation and expansion

Samples of lipoaspirate obtained from three healthy subjects (age 25–50 years) after informed consent were aseptically collected in syringes and transported to the GMP facility at controlled temperature. Soon after, the lipoaspirate was transferred into a modified Top&Bottom triple bag (Fresenius Kabi, Bad Homburg, Germany), diluted and centrifuged with sterile phosphate-buffered saline (PBS; Macopharma, Mouvaux, France) to discard red blood cells. Then the sample was digested for 40 minutes at 37°C with NB6 GMP grade collagenase (Nordmark Arzneimittel GmbH&C, Uetersen, Germany). The digestion was stopped using PBS and after centrifugation (1185 g for 30 minutes) to discard the solid fat phase, the stromal vascular fraction was seeded into a 1 layer Cell Stack (Macopharma) in a complete medium (alpha MEM, Macopharma; 10% fetal bovine serum, Australian origin, gamma irradiated, Gibco Invitrogen Corporation, Carlsbad, CA). After 24 hours, the non-adherent fraction was removed and adherent cells were cultured in complete medium. The cultures were maintained at 37°C in a humidified atmosphere containing 5% CO_2_ and the medium was changed twice a week. AD-MSC were cultured until passage 7 in order to follow the proteomic modifications well after the passages at which these cells are usually used for clinical application, that is passage 2–3. Three AD-MSC lines were seeded (3000 cells/cm^2^), passaged after detachment (TryPLE Select, Gibco Life Technologies, Italy) and counted once they reached 80% confluence. To evaluate the proliferative potential of AD-MSC, the cumulative population doublings (CPD) rate was determined using the following formula:

(1) $$PD=Log10\left(N\right)/Log10\left(2\right)$$

Where N is the ratio between the harvested cells and the seeded cells. The PD for each passage was calculated and added to the PD of the previous passages to generate data for CPD.

### Detection of telomerase activity

Telomerase activity was investigated in order to evaluate possible tumorigenic modifications in AD-MSC lines from passage 0 to passage 7 using the TeloTAGGG Telomerase PCR ELISA ^PLUS^ (Roche Applied Science, Mannheim, Germany). AD-MSC layer was dissociated by TryPLE Select and the cells were centrifuged at 300 g for 10 minutes. AD-MSC pellets (2x10^5^ cells) were resuspended in 200 μL of lysis buffer and protein extracts were obtained according to manufacturer’s protocol. Each protein extract was divided into two aliquots before performing the assay: one was used as negative control after heat inactivation of telomerase at 85°C for 10 minutes, the other one was used to evaluate the telomerase by addition of telomeric sequence. In the sample extracts telomerase was detected from the addition of telomeric repeats to the 3’ end of a biotin-labeled synthetic primer, and these elongation products were amplified by PCR. To exclude false negative results due to the presence of Taq DNA-polymerase inhibitors present in the lysates, a 216 bp internal standard (IS) was also amplified in the reaction mixture. The number of hexamers added in each sample was then evaluated by ELISA technique using TTAGGG specific probes to reveal the telomerase activity. The level of telomerase activity in each sample was determined by comparing the signal from the sample with the signal obtained using a known amount of positive control containing 0.001 amol of template DNA having the same sequence product with eight telomeric repeats. The relative telomerase activity (RTA) was calculated from different absorbances (Abs) as follows:

(2) $$RTA=\frac{\left(Absofsample-absofheat-treatedsample\right)/\left(AbsofISofthesample\right)}{\left(Absofcontroltemplate-absoflysisbuffer\right)/\left(AbsofISofthecontroltemplate\right)}$$

### Flow cytometry analysis

One x10^6^ AD-MSC at passage 4 and 7 were washed in PBS for 20 minutes at RT and incubated in the dark with the following directly conjugated mouse-anti human antibodies: CD90-FITC (Becton Dickinson, BD, San Josè, CA), CD73-APC (Miltenyi Biotec, Gladbach, Germany), CD44-FITC (BD), CD105-PE (Beckman Coulter, Fullerton, CA, USA), alpha-SMA FITC (Sigma-Aldrich), CD13-PE (BD), SSEA4-FITC (BD), CD45-PC7 (Beckman Coulter), HLA-ABC FITC (Beckman Coulter), HLA-DR PE (BD), CD34-FITC (Miltenyi), CD133-PE (Miltenyi), CD146-FITC (Miltenyi), PDGFRbeta-PE (R&D Systems, Minneapolis, MN, USA), NG2-PE (BD), NGFR-PE (BD). The isotype-matched immunoglobulins IgG1-FITC (Beckman Coulter), IgG1-PE (Beckman Coulter), IgG1-APC (Beckman Coulter) and IgG1-PC7 (Beckman Coulter) were used as negative controls under the same conditions. After staining, the cells were washed once with PBS. At least 30,000 events were acquired using the FC500 flow cytometer (Beckman Coulter) and plots were generated using the CXP analysis software (Beckman Coulter).

### Cell lysis and fractionation

AD-MSC were suspended in hypotonic buffer (10 mM HEPES, 10 mM KCl, 1.5 mM MgCl_2_) in presence of a cocktail of protease inhibitors (Complete Mini EDTA-free, Roche Diagnostics GmbH, Mannheim, Germany) and incubated for 20 minutes on ice. Cells were disrupted by fine times sonication (10 sec pulse with 10 sec pause) and then centrifuged at 15000 g for 10 minutes (4°C) to obtain two fractions, according to previously reported method with minor modifications [46]. Lysate supernatant enriched in cytoplasmic proteins was stored at −80°C, while the pellet, which was enriched in nuclear protein, was resolubilized in buffer (Urea 9 M and 10% β-mercaptoethanol) and stored at −80°C. Protein concentrations for each fraction were assessed by using Bradford protein assay according to the manufacturer’s instructions (Bio-Rad, Hercules, CA, USA).

### Protein profiling

Cytoplasmic and nuclear protein extracts were loaded, in triplicate, onto CM10 proteinchip surfaces (weak cationic exchange) as follows. Proteinchip surfaces in a Bioprocessor (Ciphergen Biosystems, Fremont, CA, USA) were pre-activated with binding buffer (low-stringency buffer 0.1 M sodium acetate pH 4) for 5 minutes. Twenty micrograms of proteins were diluted to a final volume of 100 μL with binding buffer and incubated for 45 minutes on the ProteinChips (Ciphergen Biosystems). After incubation, the chips were washed with binding buffer three times then with deionized water twice. At the end, 1 μL of matrix (half saturated sinapinic acid dissolved in 50% ACN/0.5% TFA) was added twice and arrays were analyzed with a ProteinChip Reader (PCS 4000; Ciphergen Biosystems). Each protein spot was analyzed with a setting of laser and focus mass adjusted to optimize the detection of spectral regions between 2–15 kDa and 15–30 kDa. SELDI-ToF spectra were generated by averaging 900 laser shots and calibrated using the all-in-one Protein standard II (hirudin BHVK 7.0 kDa; bovine cytochrome C 12.2 kDa; equine myoglobin 17 kDa; bovine carbonic anhydrase 29 kDa; yeast enolase 46.7; bovine albumin 66.4 kDa; and bovine IgG proteins 147.3 kDa; Ciphergen Biosystems).

### Protein purification and identification

AD-MSC cytoplasmic enriched fractions of passage 7 were pooled from each donor and were subjected to chromatographic separation to yield partially purified protein for identification. One-hundred micrograms of proteins from cell lysates were fractionated using reverse phase chromatography (Agilent mRP Hi-recovery protein column 4.6 X 50 mm) at 80°C by HPLC (Agilent 1200 Series) at 1 ml/min flow with buffer A, consisting of 0.1% TFA/water (v/v). Proteins were eluted with a binary gradient 0–100% buffer B consisting of 0.1% TFA/Acetonitrile (v/v). The gradient used was: 5 minutes in buffer A, 5.01-11 min 0–30% buffer B, 11.01-44 min 30–55% buffer B, 44.01-54 min 55–100% buffer B, the column was washed with 100% buffer B for 4 min and requilibrated with 100% buffer A for 10 minutes. The proteins were detected at 220 nm and one minute fractions were collected giving a total of 27 fractions (1 mL volume fraction). The fractions were dried under vacuum using a “speedvac” and solubilized in 50μL of ProteinChip U9 buffer (Bio-Rad). Each fraction was transferred onto Proteinchip surfaces CM10 (Ciphergen Biosystems) as previously described and analyzed by SELDI-ToF-MS. The fraction enriched for the protein of interest was digested with trypsin (sequencing grade, Sigma-Aldrich). Tryptic digestion mixture was analyzed by means of LC-MS approach, according to previously reported conditions [47].

### ImmunoAssay SELDI-TOF

Anti-S100A6 polyclonal antibody (Sigma-Aldrich) was indirectly coupled to a PS10 (Carbonyldiimidazole chemistry) array. Proteinchip surface of the PS10 array was pre-activated with 50% acetonitrile for 1 minute and 10 mM PBS pH 8 for 1 minute. Protein G (0.4 mg/mL in PBS pH 8) was incubated for 1 hour to form stable covalent bonds with Carbonyldiimidazole and unreactive sites on the chip array were blocked with an ethanolamine solution 1 M pH 8 for 30 minutes. Chip was washed twice for 5 minutes with PBS buffer pH 8 containing 0.1%(v/v) Tween 20 and then with PBS buffer 10 mM pH 8. Then, 2 μL of anti-S100A6 antibody (0.05 mg/ml) were diluted 1:10 with coupling buffer (100 mM PBS, 150 mM NaCl pH 7.5) and incubated with the chip for one hour. Chip was then washed twice for five minutes with PBS 10 mM pH 8 containing 0.1%(v/v) Tween 20, once with PBS 10 mM pH 8 and once with 10 mM HEPES pH 7. Later, 2 μg of cytoplasmic enriched fraction of passage 7 AD-MSC which had been pooled from the 3 donors was mixed with TBS-T (137 mM NaCl, 2.7 mM KCl, 25 mM Tris and 0.05%(v/v) Tween 20) and was loaded on PS10 chip. The lysate was incubated for 1.5 hours and the unbound fraction was eluted and collected. Chip was washed twice for 5 minutes with TBS pH 8 containing 0.1% (v/v) Tween 20, once with TBS pH 8 and once with 10 mM HEPES pH 7. At the end of the washes, 1μL of matrix (half saturated sinapinic acid dissolved in 50% ACN/0.5% TFA) was added twice and array was analyzed with a ProteinChip Reader. The unbound fraction was processed on Proteinchip surfaces CM10 as described previously and analyzed by SELDI-ToF-MS.

### SDS-Page/wester bloting analysis

Western blot analysis was performed on AD-MSC from each donor independently at passages P0, P1, P4, P7. At first, 5 μg of cytoplasmic lysates were added to Laemmli buffer 4X (8% SDS, 250 mM Tris–HCl pH 6.8, 40% glycerol, 20% β-mercaptoethanol and 0.1% blue of bromophenol) to obtain a 1X solution. The solution was heated to 100°C in a water bath for 5 minutes and was loaded on 18% SDS-polyacrylamide Gel (Biorad), running buffer was 25 mM Tris, 192 mM glycine 0.1% SDS, external buffer was 25 mM Tris, 192 mM glycine. Following electrophoresis proteins were transferred onto nitrocellulose membrane (Whatman, Dassel, Germany). Nitrocellulose membrane was blocked with BSA 5% and incubated overnight with primary anti-S100A6 antibody and anti-Actin antibody (Sigma). Detection was performed using anti Rabbit IgG alkaline phosphatase antibody (Sigma-Aldrich). Primary and secondary antibodies were used at dilutions of 1/1,000 and 1/5,000, respectively. Proteins were visualized by SIGMAFAST Fast Red TR/Naphthol AS-MX tablets.

### Human S100-A6 ELISA

S100A6 levels were determined using Human-S100-A6 ELISA kit (BioVendor-Laboratorni medicina a.s. Brno Czech Republic) according to the manufacturer’s instructions. ELISA was performed by using AD-MSC cytosolic extracts at dilution 1/2,500 from each donor for passages P0, P1, P4, P7.

### Data analysis

Ciphergen Express software (version 3.5, Ciphergen Biosystems) was used for pre-processing data i.e. automatic peak detection after baseline subtraction and adjustment (S/N ratio > 5, cluster mass window 0.3% peak width). The program also allows basic statistical analysis, including hierarchical clustering, group scatter plots, *P*-values (*t*-test statistics). The Mann–Whitney test was used to compare peak intensities among the AD-MSC samples (*P* value < 0.05). Additionally, multivariate analysis was carried out using relative peak intensity and was applied to the data set to identify groups and classification of different AD-MSC preparation. The coefficient of correlation was calculated including all peaks detected (53 for cytoplasmic and 82 for nuclear fraction).

A Pearson correlation coefficient was calculated for each donor in comparison with the other two at the same passage. One-way analysis of variance (ANOVA) was used to determine significance of difference in correlation with different passages. These statistical analyses were performed using Graphad Prism v 5.0 for Windows (GraphPad Software, San Diego, USA) on exported raw data that were previously converted using a square root transformation to a normal distribution [48].

## Competing interests

The authors declare that they have no competing interests.

## Authors’ contributions

EC supervised proteomic experiments and generated the manuscript, RB performed the proteomic experiments and SELDI-ToF data analysis, VP carried out AD-MSC expansion and characterization, MV performed flow cytometry analysis, LL supervised aspects of this manuscript and the experiments, AB supervised aspects of this manuscript, RG has edited the manuscript and supervised the experiments. All authors read and approved the final manuscript.

## Supplementary Material

Additional file 1 Differentially expressed peaks from different donors during subculturing passages detected in nuclear fraction by SELDI-ToF-MS. Click here for file

## References

[B1] Pittenger MF Mackay AM Beck SC Jaiswal RK Douglas R Mosca JD Moorman MA Simonetti DW Craig S Marshak DR Multilineage potential of adult human mesenchymal stem cells Science 1999 284 143 147 10.1126/science.284.5411.143 10102814

[B2] Erices A Conget P Minguell JJ Mesenchymal progenitor cells in human umbilical cord blood Br J Haematol 2000 109 235 242 10.1046/j.1365-2141.2000.01986.x 10848804

[B3] Zuk PA Zhu M Ashjian P De Ugarte DA Huang JI Mizuno H Alfonso ZC Fraser JK Benhaim P Hedrick MH Human adipose tissue is a source of multipotent stem cells Mol Biol Cell 2002 13 4279 4295 10.1091/mbc.E02-02-0105 12475952 PMC138633

[B4] Digirolamo CM Stokes D Colter D Phinney DG Class R Prockop DJ Propagation and senescence of human marrow stromal cells in culture: a simple colony-forming assay identifies samples with the greatest potential to propagate and differentiate Br J Haematol 1999 107 275 281 10.1046/j.1365-2141.1999.01715.x 10583212

[B5] Gronthos S Zannettino AC Hay SJ Shi S Graves SE Kortesidis A Simmons PJ Molecular and cellular characterisation of highly purified stromal stem cells derived from human bone marrow J Cell Sci 2003 116 1827 1835 10.1242/jcs.00369 12665563

[B6] Romanov YA Svintsitskaya VA Smirnov VN Searching for alternative sources of postnatal human mesenchymal stem cells: candidate MSC-like cells from umbilical cord Stem Cells 2003 21 105 110 10.1634/stemcells.21-1-105 12529557

[B7] D'Ippolito G Schiller PC Ricordi C Roos BA Howard GA Age-related osteogenic potential of mesenchymal stromal stem cells from human vertebral bone marrow J Bone Miner Res 1999 14 1115 1122 10.1359/jbmr.1999.14.7.1115 10404011

[B8] Bunnell BA Flaat M Gagliardi C Patel B Ripoll C Adipose-derived stem cells: isolation, expansion and differentiation Methods 2008 45 115 120 10.1016/j.ymeth.2008.03.006 18593609 PMC3668445

[B9] Lin YC Leu S Sun CK Yen CH Kao YH Chang LT Tsai TH Chua S Fu M Ko SF Wu CJ Lee FY Yip HK Early combined treatment with sildenafil and adipose-derived mesenchymal stem cells preserves heart function in rat dilated cardiomyopathy J Transl Med 2010 8 88 10.1186/1479-5876-8-88 20868517 PMC2956711

[B10] Yañez R Lamana ML García-Castro J Colmenero I Ramírez M Bueren JA Adipose tissue-derived mesenchymal stem cells have in vivo immunosuppressive properties applicable for the control of the graft-versus-host disease Stem Cells 2006 24 2582 2591 10.1634/stemcells.2006-0228 16873762

[B11] Wagner W Feldmann RE Jr Seckinger A Maurer MH Wein F Blake J Krause U Kalenka A Bürgers HF Saffrich R Wuchter P Kuschinsky W Ho AD The heterogeneity of human mesenchymal stem cell preparations-evidence from simultaneous analysis of proteomes and transcriptomes Exp Hematol 2006 34 536 548 10.1016/j.exphem.2006.01.002 16569600

[B12] Mareddy S Broadbent J Crawford R Xiao Y Proteomic profiling of distinct clonal populations of bone marrow mesenchymal stem cells J Cell Biochem 2009 106 776 786 10.1002/jcb.22088 19229859

[B13] Maddox JR Liao X Li F Niyibizi C Effects of culturing on the stability of the putative murine adipose derived stem cells markers Open Stem Cell J 2009 1 54 61 10.2174/1876893800901010054 19946473 PMC2783658

[B14] Celebi B Elçin YM Proteome analysis of rat bone marrow mesenchymal stem cell subcultures J Proteome Res 2009 8 2164 2172 10.1021/pr800590g 19323533

[B15] Zimmermann S Biniossek ML Maurer C Münzer P Pantic M Veelken H Martens UM Proteomic profiling in distinct cellular compartments of tumor cells reveals p53-dependent upregulation of S100A6 upon induction of telomere dysfunction Proteomics 2009 9 5175 5187 10.1002/pmic.200900232 19834903

[B16] Siddappa R Licht R van Blitterswijk C de Boer J Donor variation and loss of multipotency during in vitro expansion of human mesenchymal stem cells for bone tissue engineering J Orthop Res 2007 25 1029 1041 10.1002/jor.20402 17469183

[B17] Wagner W Horn P Castoldi M Diehlmann A Bork S Saffrich R Benes V Blake J Pfister S Eckstein V Ho AD Replicative senescence of mesenchymal stem cells: a continuous and organized process PLoS One 2008 3 e2213 10.1371/journal.pone.0002213 18493317 PMC2374903

[B18] Kretlow JD Jin YQ Liu W Zhang WJ Hong TH Zhou G Baggett LS Mikos AG Cao Y Donor age and cell passage affects differentiation potential of murine bone marrow-derived stem cells BMC Cell Biol 2008 9 60 10.1186/1471-2121-9-60 18957087 PMC2584028

[B19] Karystinou A Dell'Accio F Kurth TB Wackerhage H Khan IM Archer CW Jones EA Mitsiadis TA De Bari C Distinct mesenchymal progenitor cell subsets in the adult human synovium Rheumatology (Oxford) 2009 48 1057 1064 10.1093/rheumatology/kep192 19605375

[B20] Peroni D Scambi I Pasini A Lisi V Bifari F Krampera M Rigotti G Sbarbati A Galiè M Stem molecular signature of adipose-derived stromal cells Exp Cell Res 2008 314 603 615 10.1016/j.yexcr.2007.10.007 18022619

[B21] Secco M Moreira YB Zucconi E Vieira NM Jazedje T Muotri AR Okamoto OK Verjovski-Almeida S Zatz M Gene expression profile of mesenchymal stem cells from paired umbilical cord units: cord is different from blood Stem Cell Rev 2009 5 387 401 10.1007/s12015-009-9098-5 20058202 PMC2803263

[B22] Roche S Delorme B Oostendorp RA Barbet R Caton D Noel D Boumediene K Papadaki HA Cousin B Crozet C Milhavet O Casteilla L Hatzfeld J Jorgensen C Charbord P Lehmann S Comparative proteomic analysis of human mesenchymal and embryonic stem cells: towards the definition of a mesenchymal stem cell proteomic signature Proteomics 2009 9 223 232 10.1002/pmic.200800035 19142956

[B23] Issaq HJ Veenstra TD Conrads TP Felschow D The SELDI-TOF MS approach to proteomics: protein profiling and biomarker identification Biochem Biophys Res Commun 2002 292 587 592 10.1006/bbrc.2002.6678 11922607

[B24] De Bock M de Seny D Meuwis MA Chapelle JP Louis E Malaise M Merville MP Fillet M Challenges for biomarker discovery in body fluids using SELDI-TOF-MS J Biomed Biotechnol 2010 2010 906082 20029632 10.1155/2010/906082 PMC2793423

[B25] Mazzatti DJ Pawelec G Longdin R Powell JR Forsey RJ SELDI-TOF-MS ProteinChip array profiling of T-cell clones propagated in long-term culture identifies human profilin-1 as a potential bio-marker of immunosenescence Proteome Sci 2007 5 7 10.1186/1477-5956-5-7 17550585 PMC1892543

[B26] McLean K Wu Y Gan BS O'Gorman DB An alternative kinase activity assay for primary cultures derived from clinical isolates Clin Invest Med 2009 32 E84 E94 19331809 10.25011/cim.v32i2.6025

[B27] Reddy G Dalmasso EA SELDI ProteinChip® Array Technology: Protein-Based Predictive Medicine and Drug Discovery Applications J Biomed Biotechnol 2003 2003 237 241 10.1155/S1110724303210020 14615631 PMC514271

[B28] Pilgaard L Lund P Duroux M Lockstone H Taylor J Emmersen J Fink T Ragoussis J Zachar V Transcriptional signature of human adipose tissue-derived stem cells (hASCs) preconditioned for chondrogenesis in hypoxic conditions Exp Cell Res 2009 315 1937 1952 10.1016/j.yexcr.2009.01.020 19331821

[B29] Donato R Functional roles of S100 proteins, calcium-binding proteins of the EF-hand type Biochim Biophys Acta 1999 1450 191 231 10.1016/S0167-4889(99)00058-0 10395934

[B30] Breen EC Tang K Calcyclin (S100A6) regulates pulmonary fibroblast proliferation, morphology, and cytoskeletal organization in vitro J Cell Biochem 2003 88 848 854 10.1002/jcb.10398 12577318

[B31] Okazaki K Niki I Iino S Kobayashi S Hidaka H A role of calcyclin, a Ca(2+)-binding protein, on the Ca(2+)-dependent insulin release from the pancreatic beta cell J Biol Chem 1994 269 6149 6152 8119959

[B32] Leśniak W Słomnicki ŁP Filipek A S100A6 - new facts and features Biochem Biophys Res Commun 2009 390 1087 1092 10.1016/j.bbrc.2009.10.150 19891957

[B33] De Petris L Orre LM Kanter L Pernemalm M Koyi H Lewensohn R Lehtiö J Tumor expression of S100A6 correlates with survival of patients with stage I non-small-cell lung cancer Lung Cancer 2009 63 410 417 10.1016/j.lungcan.2008.06.003 18620780

[B34] Nowotny M Spiechowicz M Jastrzebska B Filipek A Kitagawa K Kuznicki J Calcium-regulated interaction of Sgt1 with S100A6 (calcyclin) and other S100 proteins J Biol Chem 2003 278 26923 26928 10.1074/jbc.M211518200 12746458

[B35] Kuźnicki J Kordowska J Puzianowska M Woźniewicz BM Calcyclin as a marker of human epithelial cells and fibroblasts Exp Cell Res 1992 200 425 430 10.1016/0014-4827(92)90191-A 1572406

[B36] Leśniak W Swart GW Bloemers HP Kuźnicki J Regulation of cell specific expression of calcyclin (S100A6) in nerve cells and other tissues Acta Neurobiol Exp (Wars) 2000 60 569 575 11200185 10.55782/ane-2000-1377

[B37] Mandinova A Atar D Schäfer BW Spiess M Aebi U Heizmann CW Distinct subcellular localization of calcium binding S100 proteins in human smooth muscle cells and their relocation in response to rises in intracellular calcium J Cell Sci 1998 111 2043 2054 9645951 10.1242/jcs.111.14.2043

[B38] Tsoporis JN Izhar S Parker TG Expression of S100A6 in cardiac myocytes limits apoptosis induced by tumor necrosis factor-alpha J Biol Chem 2008 283 30174 30183 10.1074/jbc.M805318200 18753141 PMC2662078

[B39] Lorenz S Eszlinger M Paschke R Aust G Weick M Führer D Krohn K Calcium signaling of thyrocytes is modulated by TSH through calcium binding protein expression Biochim Biophys Acta 2010 1803 352 360 10.1016/j.bbamcr.2010.01.007 20083144

[B40] Ito M Kizawa K Expression of calcium-binding S100 proteins A4 and A6 in regions of the epithelial sac associated with the onset of hair follicle regeneration J Invest Dermatol 2001 116 956 963 10.1046/j.0022-202x.2001.01369.x 11407987

[B41] Silva WA Jr Covas DT Panepucci RA Proto-Siqueira R Siufi JL Zanette DL Santos AR Zago MA The profile of gene expression of human marrow mesenchymal stem cells Stem Cells 2003 21 661 669 10.1634/stemcells.21-6-661 14595126

[B42] Jeong JA Hong SH Gang EJ Ahn C Hwang SH Yang IH Han H Kim H Differential gene expression profiling of human umbilical cord blood-derived mesenchymal stem cells by DNA microarray Stem Cells 2005 23 584 593 10.1634/stemcells.2004-0304 15790779

[B43] Ghezzo F Lauret E Ferrari S Baserga R Growth factor regulation of the promoter for calcyclin, a growth-regulated gene J Biol Chem 1988 263 4758 4763 2832406

[B44] Rahimi F Hsu K Endoh Y Geczy CL FGF-2, IL-1beta and TGF-beta regulate fibroblast expression of S100A8 FEBS J 2005 272 2811 2827 10.1111/j.1742-4658.2005.04703.x 15943814

[B45] Russell KC Phinney DG Lacey MR Barrilleaux BL Meyertholen KE O'Connor KC In vitro high-capacity assay to quantify the clonal heterogeneity in trilineage potential of mesenchymal stem cells reveals a complex hierarchy of lineage commitment Stem Cells 2010 28 788 798 10.1002/stem.312 20127798

[B46] Marshall J Krump E Lindsay T Downey G Ford DA Zhu P Walker P Rubin B Involvement of cytosolic phospholipase A2 and secretory phospholipase A2 in arachidonic acid release from human neutrophils J Immunol 2000 164 2084 2091 10657662 10.4049/jimmunol.164.4.2084

[B47] Regonesi ME Del Favero M Basilico F Briani F Benazzi L Tortora P Mauri P Dehò G Analysis of the Escherichia coli RNA degradosome composition by a proteomic approach Biochimie 2006 88 151 161 10.1016/j.biochi.2005.07.012 16139413

[B48] Goyak KM Johnson MC Strom SC Omiecinski CJ Expression profiling of individual variability following xenobiotic exposures in primary human hepatocyte cultures Toxicol Appl Pharmacol 2008 231 216 224 10.1016/j.taap.2008.04.024 18559280 PMC2610447

