# Supplementary material for: Changes in the proteomic profile of adipose tissue-derived mesenchymal stem cells during passages
Source: Proteome Sci. 2012 Jul 24;10:46. doi: 10.1186/1477-5956-10-46 (PMC3499380; doi:10.1186/1477-5956-10-46)
Supplement: Additional file 1 — Differentially expressed peaks from different donors during subculturing passages detected in nuclear fraction by SELDI-ToF-MS. [file 1477-5956-10-46-S1.pdf]

Additional file 1. Differentially expressed peaks from different donors during subculturing passages detected in nuclear fraction by SELDI-ToF-MS.

Mass/charge ratio	Intensity average	p-Value	Mass/charge ratio	Intensity average	p-Value
8163,4	20,9	0,01138	6839,8	18,3	0,11699
9515,4	22,0	0,01398	4946,3	45,1	0,11952
7960,7	33,7	0,01639	4316,7	98,9	0,12210
11651,5	156,7	0,01792	8280,1	10,6	0,13517
11727,5	116,4	0,01810	4197,2	13,6	0,14882
11828,1	33,7	0,01910	4729,7	12,7	0,15006
14706,0	18,1	0,02017	6725,0	16,4	0,15640
6280,4	21,3	0,02046	4058,3	37,4	0,15965
8089,2	58,1	0,02097	9085,4	36,5	0,16230
9229,5	18,6	0,02335	6798,1	19,0	0,16363
4971,4	82,2	0,02369	3525,0	9,9	0,17046
7884,4	87,1	0,02499	6330,7	14,2	0,17255
14779,6	18,4	0,02598	11072,2	70,0	0,17255
6303,5	18,9	0,02807	6370,3	12,9	0,20019
12649,8	5,5	0,02960	3997,3	14,3	0,20663
12276,8	16,0	0,03090	6596,1	18,4	0,21491
5830,8	46,6	0,03226	6351,2	16,0	0,22086
3659,2	3,7	0,03515	3236,1	18,0	0,25075
5795,5	19,6	0,03532	8565,5	46,2	0,27035
5654,5	80,1	0,03583	10167,0	92,2	0,29111
9153,6	32,7	0,04129	7014,9	11,2	0,29325
3362,7	21,4	0,04148	3582,6	3,6	0,29432
6981,4	14,4	0,04148	3751,9	9,4	0,29648
10271,8	31,4	0,04227	9742,3	7,5	0,30522
4897,6	15,7	0,04472	7468,2	4,1	0,30633
12496,7	10,8	0,04685	13778,4	29,2	0,35793
7391,5	9,0	0,05047	12170,5	9,4	0,36415
5361,7	35,0	0,05118	9697,8	3,9	0,38067
6652,8	22,8	0,05214	9293,5	7,1	0,38325
5633,7	54,2	0,05287	11305,5	70,2	0,39766
7932,4	25,3	0,05744	7413,0	6,6	0,42197
10352,9	19,7	0,05744	7004,2	16,1	0,42749
10840,7	28,4	0,05877	10091,0	130,8	0,43726
13437,5	5,3	0,06041	14004,8	24,1	0,44148
8960,7	32,2	0,06068	7785,1	13,0	0,46876
7815,7	15,6	0,07018	11347,5	39,7	0,56013
6684,2	17,3	0,07146	5283,6	5,0	0,58395
5965,2	24,4	0,07542	4243,2	10,4	0,59356
7448,3	9,5	0,10056	8361,0	9,6	0,64695
6664,9	18,0	0,10920	7678,0	5,1	0,77670
11149,3	54,6	0,11352	6893,8	15,8	0,91075
